# ProOvErlap: Assessing feature proximity/overlap and testing statistical significance from genomic intervals

**DOI:** 10.1016/j.jbc.2025.110209

**Published:** 2025-05-08

**Authors:** Nicolò Gualandi, Alessio Bertozzo, Claudio Brancolini

**Affiliations:** Department of Medicine, Università degli Studi di Udine, Udine, Italy

**Keywords:** promoters, enhancers, ChIP-seq, RNA-seq, ATAC-seq, gene expression, bioinformatics, leyomioma, BRD4, SP1, H3K27ac, H3K9ac, H3K27me3

## Abstract

Feature overlap is a critical concept in bioinformatics and occurs when two genomic intervals, usually represented as BED files, are located in the same genomic regions. Instead, feature proximity refers to the spatial proximity of genomic elements. For example, promoters typically overlap or are close to the genes they regulate. Overlap and proximity are also important in epigenetic studies. Here, the overlap of regions enriched for specific epigenetic modifications or accessible chromatin can elucidate complex molecular phenotypes. Consequently, the ability to analyze and interpret feature overlap and proximity is essential for understanding the biological processes that contribute to a given phenotype. To address this need, we present a computational method capable of analyzing data represented in the BED format. This method aims to quantitatively assess the degree of proximity or overlap between genomic features and to determine the statistical significance of these events in the context of a nonparametric randomization test. The aim is to understand whether the observed state differs from what would be expected by chance. The method is designed to be easy to use, requiring only a single command line to run, allowing straightforward overlap and proximity analysis. It also provides clear visualizations and publication-quality figures. In conclusion, this study highlights the importance of feature overlap and proximity in epigenetic studies and presents a method to improve the systematic assessment and interpretation of these features. We propose a new resource for identifying biologically significant interactions between genomic features in both healthy and disease states.

Feature proximity/overlap is a crucial concept in bioinformatics that plays a central role in the analysis and interpretation of genomic data (1). In genomic studies, the identification of nearby or overlapping genomic features can reveal crucial biological regulations and functional relationships in the mechanisms that control gene expression, protein interactions, and cellular processes (2, 3, 4). In this scenario, understanding the one-dimensional spatial relationships between genomic elements is essential for identifying colocalization patterns, regulatory regions, and possible functional interactions between different types of genomic features.

Genomic features such as genes, regulatory elements, enhancers, and transcription factor–binding sites, among others, play a central role in biological processes and disease mechanisms (5, 6). These features are typically studied in the context of next-generation sequencing (NGS) technologies such as RNA-seq, chromatin immunoprecipitation sequencing (ChIP-seq), and assay for transposase-accessible chromatin by sequencing (ATAC-seq) (7).

Many of the NGS techniques produce genomic intervals as a result, which can represent features such as genes, open chromatin regions, or regions bound by specific proteins. These features are usually represented in a BED file—a standard bioinformatics file format that contains information about the chromosome, the start and end coordinates, the name of the feature, the score, and the strand direction. Often these experiments include comparisons that identify subsets of features that are relevant to the experimental context. For example, they may highlight regions that are differentially bound by a particular protein when comparing healthy and diseased states, or a list of differentially expressed genes under different conditions, or a set of open chromatin regions that only appear after a particular treatment. Identifying significant overlap or proximity between such features under different experimental conditions is critical to understanding their biological significance (8).

Indeed, overlapping or close features can provide important insights into functional relationships between genes and regulatory elements that may contribute to disease pathogenesis or cellular responses to environmental stimuli as well as under physiological conditions (9, 10). For example, overlapping or close features between gene expression profiles and regulatory regions can provide important insights into the regulatory networks that control gene activity (10). In ChIP-seq experiments, where protein–DNA interactions are mapped, overlapping features may indicate that certain transcription factors or chromatin-modifying proteins bind to the same regions of the genome (11). Identifying these overlaps is critical to understand how regulatory elements interact to regulate gene expression. ATAC-seq, which provides insight into chromatin accessibility, can also benefit from analyzing proximity/overlap of features. Accessible chromatin regions, often associated with active gene promoters, may overlap or lie in proximity to specific histone modifications or transcription factor–binding sites (12, 13). Analysis of such scenarios can shed light on how chromatin structure dynamically changes in both physiological and disease states and may provide a key strategy for identifying functional associations, regulatory networks, and molecular mechanisms that control cell behavior (6).

The ability to detect proximity/overlap of features is not only descriptive but also requires statistical methods to determine whether the observed overlap is biologically meaningful or whether it could have occurred by chance (1). Random overlap is a potential problem, especially when working with large genomic datasets where the large number of features can lead to seemingly significant overlaps that are in fact random events. Therefore, statistical tools that assess the degree of overlap between two feature sets are crucial to filter out spurious associations and identify those that are likely to be biologically relevant.

The randomization test is one such statistical approach that can be used to quantify the significance of proximity/overlap between genomic features (14). In this method, genomic intervals are randomly selected multiple times to generate a distribution of expected proximity/overlap under the null hypothesis that there is no biological interaction between the trait sets. The observed closeness/overlap is then compared to this null distribution to determine if it exceeds the expected overlap by chance.

Here we present a computational tool to assess the overlap and/or proximity of features in genomic datasets represented as BED files (15). The tool uses the Python library pybedtools to identify overlaps and compute distances between genomic regions. The tool integrates randomization and permutation tests to assess the statistical significance of overlap or closeness. The algorithm has been designed to be intuitive to use, making it accessible to researchers with varying levels of bioinformatics skills. By providing clear visualizations and output tables, the tool facilitates interpretation of results and allows researchers to hypothesize about gene regulation, chromatin remodeling, and other biological processes.

## Results

### ProOvErlap effectively identifies significant enrichment and proximity in synthetic datasets

To evaluate the tool in a controlled environment, we first generate synthetic data: i) a BED input file with 500 nonoverlapping genomic intervals; ii) a BED background file with 2000 features containing the 500 genomic intervals from the input file together with 1500 nonoverlapping randomly selected regions. Finally, (iii) a target BED file was created with 5000 nonoverlapping genomic intervals, of which 125 regions overlapped with the regions from the input file. This setup was intended to achieve significant overlap, with 25% of the regions in the input file (125 out of 500) overlapping the target file, while only 6.25% of the regions (125 out of 2000) from the background file overlapped the target file. In this way, we expected to observe a significant enrichment of the input regions compared to the background. The analysis was performed with the following command:

“*python3 prooverlap.py --mode intersect --input Input.bed --target Target.bed --background Background.bed --randomization 100 --orientation strandless --outfile Test_intersect.txt*”.

**Figure 1:**
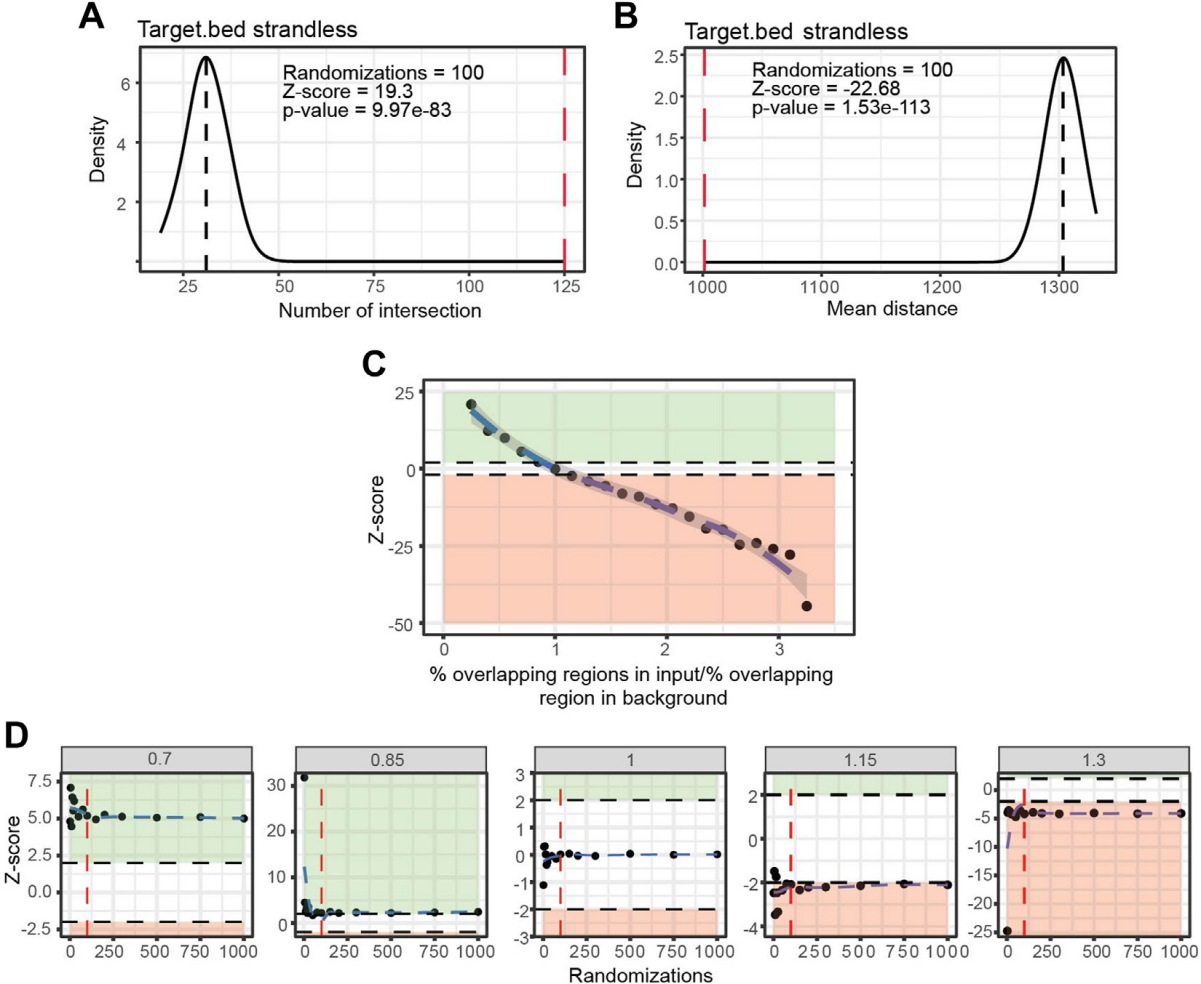
**ProOvErlap testing in synthetic datasets**. *A*, density plot showing the distribution of the number of intersections from the random sampling compared to the real value (*red dashed line*). The *black dashed line* represents the mean value of the random number of overlaps with respect to the target regions over N randomizations (here N = 100). *B*, density plot showing the distribution of the mean distance from the random sampling compared to the real value (*red dashed line*). The *black dashed line* represents the mean of the random mean value distances to the target regions over N randomizations (here N = 100). *C*, correlation between the ratio of percentage overlap between the input and background files (the percentage overlap represents the percentage of regions that overlap with the target relative to the total number of regions in the input or background file) and the Z-score (randomizations = 100). Regions highlighted in *green* represent Z-scores > 2, while regions highlighted in *red* represent Z-scores < 2. *D*, correlation between the number of randomizations and the Z-score. Each facet represents a different ratio of overlap percentages between the input and background files (the overlap percentage represents the percentage of regions in overlap with the target relative to the total number of regions in the input or background file). Regions highlighted in *green* represent Z-scores > 2, while regions highlighted in *red* represent Z-scores < 2. The vertical *dashed red line* represents N = 100 randomizations.

Table 1 shows the results of the test. The results confirmed a significant enrichment of the overlap between the input and target regions, as shown in Figure 1*A* (Z-score 19.3), compared to the background.

**Table 1:** Results of an analysis in which the number of features in the input.bed file that overlap with the target file is compared with the mean value of 100 randomizations from the background.bed file

Z-Score	Type	*p*-value	Target	Real	Random	sd
19.3	strandless	9.97e-83	Target.bed	125	31.01	4.87

The “Z-score” column contains the Z-score of the comparison. The “Type” column indicates the type of test used (in this case, “strandless”), but other possible types are “concordant,” “discordant,” “AT_GC,” and/or “length.” The “*p*-value” column indicates the *p*-value, while the “Target” column lists the name of the target file against which the enrichment is tested. The "Real" column shows the actual number of intersections between the input and the target bed files, while the “Random” column shows the mean number of intersections obtained from the unbiased background distribution after N randomizations (N = 100). The “SD” column indicates the standard deviation of the unbiased distributions. If multiple tests or target files are involved, each test or target file is shown in a separate row.

We then performed a similar analysis with the "closest" mode. We generated 500 features with a mean distance of 1000 ± 200 nt to the target sequences (where the target file contained 5000 features). In addition to the 500 input features, the background file contained 1500 randomly generated features with a mean distance of 1400 ± 600 nt to the closest feature in the target file, for a total of 2000 regions.

We run the analysis with the following command:

“*python3 prooverlap.py --mode closest --input Input.bed --target Target.bed --background Background.bed --randomization 100 --orientation strandless --outfile Test_closest.txt --exclude_ov* ”

Table 2 shows the results of the test. As shown in Figure 1*B*, the tool detected a significant negative enrichment (Z-score = −22.68), with the features of the input file being significantly closer to the features of the target file compared to the background.

**Table 2:** Results of an analysis comparing the mean distance of features in the input.bed file to the nearest feature in the target file with the mean value of 100 randomizations from the background.bed file

Z-Score	Type	*p*-value	Target	Real	Random	sd
−22.68	strandless	1.53e-113	Target.bed	1001.37	1303.34	13.32

The “Z-score” column contains the Z-score of the comparison. The “Type” column indicates the type of test used (in this case, “strandless”), but other possible types are “concordant,” “discordant,” “AT_GC,” and/or “length.” The “*p*-value” column indicates the *p*-value, while the “Target” column lists the name of the target file against which enrichment is tested. The “Real” column shows the mean distance between the input and the closest feature in the target.bed files, while the “Random” column shows the mean distance obtained from the unbiased background distribution after N randomizations (N = 100). The “SD” column indicates the standard deviation of the unbiased distributions. If multiple tests or target files are involved, each test or target file is shown in a separate column.

To test the sensitivity of this approach, we randomly generated background files with increasing percentages of overlapping regions with the target file (6.25%, 13.75%, 17.50%, 21.25%, 25.00%, 28.75%, 32.50%, 36.25%, 40.00%, 43.75%, 47.50%, 51.25%, 55.00%, 58.75%, 62.50%, 66.25%, 70.00%, 73.75%, 77.50%), while the overlap percentage for the input file was kept at 25%. Figure 1*C* shows the correlation (randomization = 100) between the obtained Z-scores and the ratio of the percentage overlap between the input and background files with respect to the target. As expected, when the percentage of overlap between the input and background files was similar or equal (percentage overlap ratio = 1), the Z-score was close to zero, indicating no enrichment. When the percentage of overlap changed, we observed significant Z-scores (|Z-score| > 2, corresponding to a *p*-value of < 0.04 from a two-tailed Gaussian distribution), indicating enrichment or depletion of overlap between the input and target files compared to background. When the overlap ratio between the input and background files was between 0.85 and 1.15, the Z-score was close to the significance threshold (|Z-score| ∼ 2, which corresponds to a *p*-value of ∼ 0.04 from a two-tailed Gaussian distribution). This indicates that under the hypothesis tested, the tool classifies differences up to a relative enrichment of 15% between the input and background files with respect to the target as significant. However, it should be noted that the detection of significance may also depend on various other factors, such as the number of regions in the input and background datasets and the variability within these datasets (explained by the standard deviation). In other words: while an enrichment of 15% may be identified as significant under current conditions, the significance threshold could change if these factors change.

To assess the impact of the number of randomizations, we tested different randomization ranges from 2 to 1,000, using overlap ratios between 0.7 and 1.3, as these overlap ratios were identified as borderline and provide a good opportunity to test for uncertain situations under the current conditions. As can be seen in Figure 1*D*, Z-scores were less stable with less than 100 randomizations and were subject to more random fluctuations, but stabilized after 100 randomizations, suggesting that 100 randomizations are the minimum required for reliable results. Increasing the number of randomizations beyond 100 had no significant effect on the results, although sensitivity could be affected when testing for smaller differences in overlap, in which case more randomizations should be used.

Finally, a set of synthetic BED files with an increasing number of regions was generated to evaluate ProOvErlap in terms of running time and memory consumption. The input BED files contained a series of genomic intervals between 500 and 10,000, increasing in increments of 500. The corresponding background BED file contained twice the number of regions as the input BED file, while the target BED file contained three times the number of features present in the input BED file. The files were created so that 25% of the features in the input file overlapped with the target BED file. We then performed a standard analysis with ProOvErlap in intersect and closest mode, with “strandless” orientation, single-threaded execution, and 100 randomizations. Our results show that both execution time and memory consumption increase linearly with the number of input regions (Fig. S1, *A*–*D*). The results may vary when multithreading is used, especially for very large files that exceed a certain number of regions, when the option to generate a randomized background is enabled, and especially when a high number of randomizations are used, as these can easily be distributed to different processors in parallel. Multithreading can improve performance, especially with extremely large files or a high number of randomizations. When working with high number of randomizations and multiple threads are available, the time required for analysis can generally be improved.

### Comparison of ProOvErlap with similar tools for analyzing overlap enrichment

To compare the results obtained with ProOvErlap with those of similar tools, we selected four alternative methods that can perform similar operations. The selected tools were LOLA, regioneR, CoBind, and Bedtools Fisher (16, 17, 18, 19). All tools were tested with their default parameter settings as specified in their respective manuals. The input set consisted of randomly generated BED files with an increasing percentage of overlap between the input and target files. The percentage of overlap started at 0% (no overlap) and was increased in five increments to 100% (all input features overlapped with at least one target feature), generating a total of 20 different test cases. All BED files were sorted by position using bedtools sort (19).

CoBind was tested using the “cooccur” function:

“cobind.py cooccur Input_sorted.bed Target_sorted.bed Background_sorted.bed Bedtools”

BedTools Fisher was executed with the following command:

“bedtools fisher -a Input_sorted.bed -b Target_sorted.bed -g Background_sorted.bed”

while regioneR and LOLA were run within R using their respective functions in R.

For regioneR:

“overlapPermTest(A = Input_sorted.bed, B = Target_sorted.bed, ntimes = 100, universe = Background_sorted.bed)”

for LOLA:

“runLOLA(Input_sorted.bed, Background_sorted.bed, Target_sorted.bed, cores = 1)”

A high correlation was observed between ProOvErlap and all other tested tools (Fig. S2, *A*–*E*). ProOvErlap showed a near-perfect correlation (Pearson correlation coefficient = 0.99) with both LOLA and CoBind. The Pearson correlation coefficient was 0.92 for Bedtools Fisher and 0.83 for regioneR (Fig. S2*E*). Nevertheless, the results of regioneR remained largely consistent, with a correlation coefficient of 0.83 and a statistical significance of *p*-value = 3.1 × 10^−6^. In summary, ProOvErlap provides results comparable to all tested tools.

### Expressed genes are located closer to open chromatin regions than nonexpressed genes

After proving the reliability of ProOvErlap in a synthetic model, we tested it in a real biological question. Actively transcribed genes are generally located near open chromatin regions, while nontranscribed or poorly transcribed genes are located further away (20, 21). This proximity of an open chromatin state is essential for transcription factors to reach the DNA sequence and initiate transcription, a process that involves many other proteins, including RNA polymerase II for several coding and noncoding genes (12, 20, 22). To test ProOvErlap, we retrieved matched RNA-seq and ATAC-seq data from the ENCODE project for the human lymphoblast cell line derived from the bone marrow K562 (4, 23). To consistently identify expressed genes and open chromatin regions, we extracted conserved IDR-thresholded peaks in BED file format. Expressed genes were defined as those whose FPKM was greater than 1 in both replicates (12,456 genes), while all annotated genes were used as background (57,400 genes). We performed both “closest” and “intersect” analyses using all annotated genes as background to test whether expressed genes were systematically closer to or overlapped with ATAC-seq peaks compared to nontranscribed genes.

The closest analysis was performed with the following command line:

“Python3 prooverlap.py –mode closest –input Expressed_genes.bed –target ENCFF695IGF.bed –background All_genes.bed –randomization 100 –orientation strandless –outfile Test_closest_ATAC_bg_allgenes.txt”

The intersect analysis was performed with the following command line:

“python3 prooverlap.py --mode intersect --input Expressed_genes.bed --target ENCFF695IGF.bed --background All_genes.bed --randomization 100 --orientation strandless --outfile Test_intersect_ATAC_bg_allgenes.txt”

**Figure 2:**
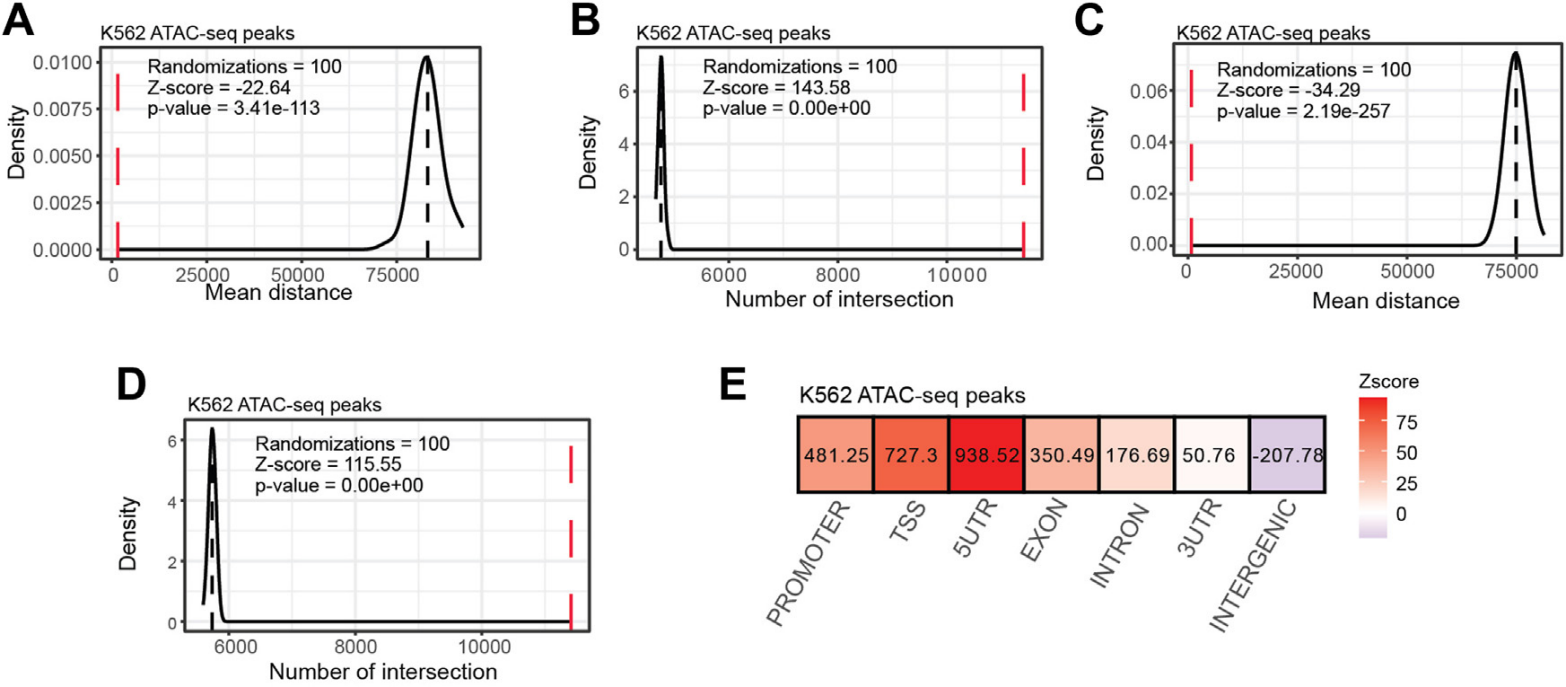
**The use of ProOvErlap to find correlations between gene expression and chromatin accessibility using ENCODE data.***A*, density plot showing the distribution of the mean distance from the random sample compared to the real value (*red dashed line*, mean distance of expressed genes from the nearest ATAC peak). The *black dashed line* represents the mean of the distances of the random genes from the nearest ATAC peak across N randomizations (here N = 100). *B*, density plot showing the distribution of the number of intersections from the random sample compared to the real value (*red dashed line*, number of expressed genes in overlap with at least one ATAC peak). The *black dashed line* represents the mean of the number of overlaps of random genes with the ATAC peaks over N randomizations (here N = 100). *C*, density plot showing the distribution of the mean distance from the random sample compared to the real value (*red dashed line*, mean distance of expressed genes to the ATAC peak). The *black dashed line* represents the mean of the distances of the randomly generated regions from the nearest ATAC peak over N randomizations (here N = 100). A custom background was generated with the flag “-generate_bg” to create a background adapted to the chromosome frequency and length. *D*, density plot showing the distribution of the number of intersections from the random sample compared to the real value (*red dashed line*, number of expressed genes in overlap with at least one ATAC peak). The *black dashed line* represents the mean of the number of overlaps of randomly generated regions with the ATAC peaks across N randomizations (here N = 100). A custom background was generated using the “—generate_bg” flag to create a chromosome frequency and length matched background. *E*, heatmap of Z-scores comparing the localization of ATAC-seq peaks overlapping with expressed genes over different genomic regions, including intergenic regions, untranslated regions (UTRs), transcription start sites (TSS), promoters, exons, and introns.

Additionally, we performed the same analyses with a randomly generated background for comparison purposes by adding the option “--generate_bg” to the previous command. As shown in Figure 2*A*, the “closest” analysis shows that the expressed genes are significantly closer to the ATAC-seq peaks (mean distance = 1537.62 and Z-score = −22.64) with respect to nonexpressed genes. Similar results were obtained using the “intersect” approach, which highlighted 11,411 (out of 12,456) genes that overlapped with at least one ATAC-seq peak (Z-score with respect to nonexpressed genes = 143.58, Fig. 2*B*). For comparison, we repeated the closest and intersect analyses with a random background corresponding to the chromosome frequency and length distribution of the expressed genes. These analyses yielded comparable results, with expressed genes closer to or overlapping the ATAC-seq peaks than expected by chance (closest Z-score = −34.29, intersect Z-score = 111.55, Fig. 2, *C* and *D*).

Furthermore, we observed similar results when using the random background, suggesting that while a coherent background model leads to more precise and accurate results, the use of a random background adjusted for chromosome frequency and length also provides reliable results when a background file is not available. Taken together, these results confirm that expressed genes are significantly closer to or overlap with open chromatin regions compared to unexpressed genes (20, 21, 24, 25) and the method used is effective in determining the association between expressed genes and accessible chromatin regions.

To investigate the localization of ATAC-seq peaks overlapping with expressed genes with respect to different genomic structures, we analyzed their distribution across intergenic regions, promoters, transcription start sites (TSSs), UTRs, exons, and introns. We used the “–GenomicLocalization” option to test the genomic localization of the detected overlaps with respect to genomic structures. The list of ATAC-seq peaks was used as input and the list of expressed genes (genes with FPKM > 1) as target. In addition, we generated a random chromosomal background matched to the chromosomal frequency and length as a control (option “--generate_bg”). As shown in Figure 2*E*, we observed a significantly positive Z-score for all tested gene regions (promoters, TSSs, UTRs, exons, and introns). Especially promoters, 5′-UTRs, and TSS showed a stronger enrichment than introns, exons, and 3′-UTRs. This indicates that ATAC-seq peaks are more frequently localized at the beginning of genes, possibly contributing to transcription initiation. Conversely, we found a depletion of ATAC-seq peaks in intergenic regions, further supporting the spatial association between ATAC-seq peaks and gene-related regions, particularly TSSs, promoter regions, and 5′-UTRs, all of which are located at the start of genes. These results were expected and confirm the effectiveness of ProOvErlap.

### ProOvErlap and the genome-wide analysis of BRD4 and SP1 colocalization with histone marks: linking gene expression to epigenetic markers

To further demonstrate the simplicity and efficacy of ProOvErlap, data from the ENCODE repository for the K562 cell line were again used (4). ChIP-seq data for three histone markers: H3K27ac (ENCFF864OSZ), H3K27me3 (ENCFF801AHF), and H3K9ac (ENCFF148UQI), together with polyA + RNA-seq gene expression data (ENCFF556ISR) were downloaded. In addition, ChIP-seq data for the histone acetylation reader BRD4 (ENCFF130JVF) and the transcription factor SP1 (ENCFF553GPK) were obtained.

H3K27ac marks active enhancers and promoters of expressed genes, whereas H3K27me3 is an exclusively repressive marker that is found at repressed promoter regions (26, 27, 28, 29, 30, 31, 32, 33, 34). To test how ProOvErlap performs when examining the relationships between chromatin status/epigenetics and gene expression, we created three BED files based on the RNA-seq gene expression matrix, representing different levels of gene expression around the TSSs with a genomic range of −3000 to +3000 bases. The categories were defined as follows: very low expression (<1 TPM), low expression (>1 TPM & < 10 TPM), medium-high expression (>10 TPM). We then used ProOvErlap to examine the enrichment of H3K27ac and H3K27me3 signals in these regions, using the whole genome’s TSS regions as background. The analysis was performed with the following commands:

- For very low expression genes:

“python3 prooverlap.py --input TSS_Very_low_expr.bed --targets ENCFF864OSZ_ H3K27ac.bed, ENCFF801AHF_H3K27me3.bed --background All_TSS.bed --randomization 100 --mode intersect --genome genome.fa --orientation strandless --outfile TSS_Very_low_expr.txt”

- For Low expression genes:

“python3 prooverlap.py --input TSS_Low_expr.bed --targets ENCFF864OSZ_ H3K27ac.bed, ENCFF801AHF_H3K27me3.bed --background All_TSS.bed --randomization 100 --mode intersect --genome genome.fa --orientation strandless --outfile TSS_Low_expr.txt”

- For Medium to high expression genes:

“python3 prooverlap.py --input TSS_Medium_High_expr.bed --targets ENCFF864OSZ_ H3K27ac.bed, ENCFF801AHF_H3K27me3.bed --background All_TSS.bed --randomization 100 --mode intersect --genome genome.fa --orientation strandless --outfile TSS_Medium_High_expr.txt”

**Figure 3:**
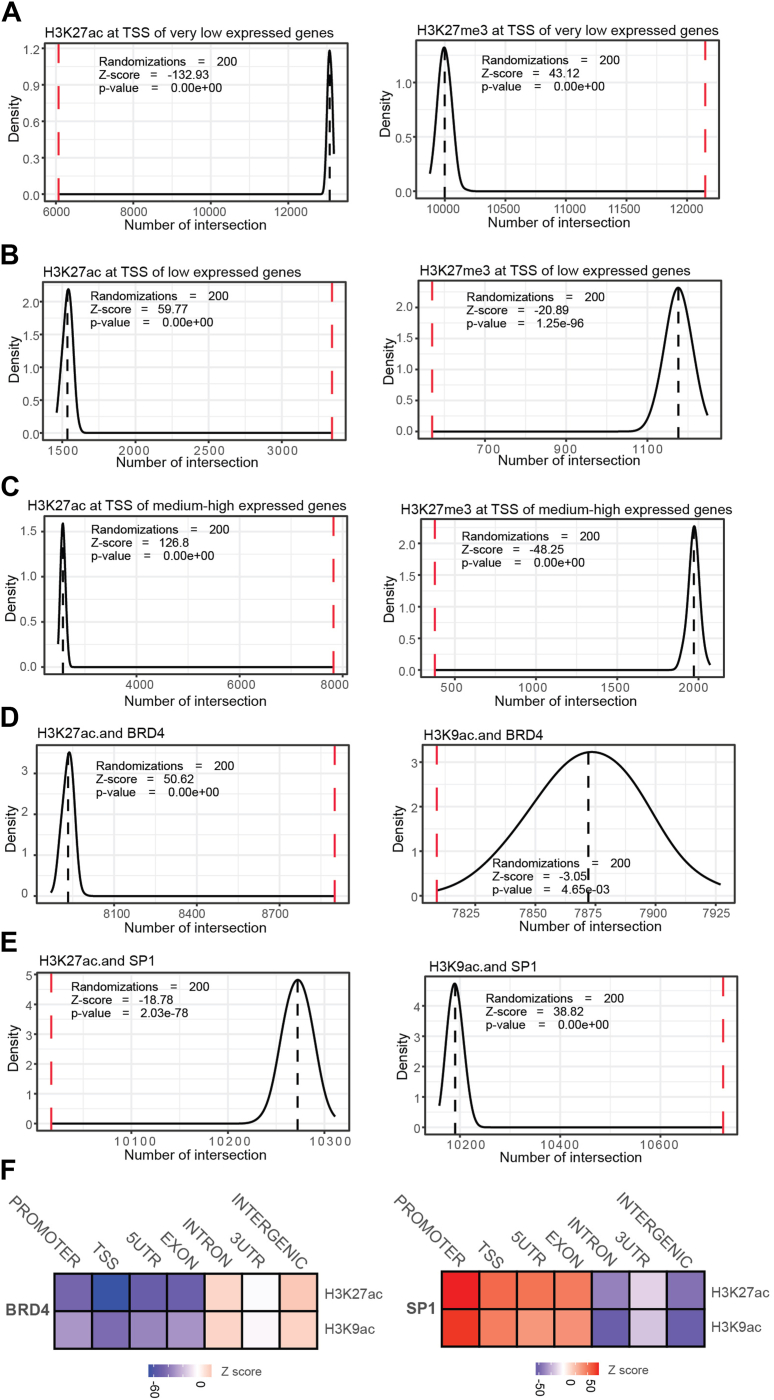
**ProOvErlap and the epigenetic correlations with gene expression levels.***A*, density plot showing the distribution of the mean distance from the random sampling compared to the real value (*red dashed line*: observed value overlap, *black dashed line*: expected number of overlaps from the randomization, number of randomizations = 100). Overlap between TSSs of very low expressed genes with H3K27ac peaks (*left*) and H3K27me3 (*right*). *B*, as in (*A*), but overlaps are between TSSs of low expressed genes with H3K27ac peaks (*left*) and H3K27me3 (*right*). *C*, as in (*A*), but overlaps are between TSSs of medium-high expressed genes with H3K27ac peaks (*left*) and H3K27me3 (*right*). *D*, density plot showing the distribution of the mean distance from the random sampling compared to the real value (*red dashed line*: observed value overlap, *black dashed line*: expected number of overlaps from randomization, N randomizations = 100). Overlap between BRD4 peaks with H3K27ac peaks (*left*) and H3K9ac (*right*). *E*, as in (*D*), but overlap between SP1 peaks with H3K27ac peaks (*left*) and H3K9ac (*right*). *F*, heatmaps showing the Z-score of BRD4 (*left*) and SP1 (*right*) enrichment of overlaps with targets across different genomic regions.

The results of the analysis are shown in Figure 3. For very-low expressed genes (TPM < 1), the TSS of these genes shows a clear reduced overlap with H3K27ac and higher overlap with H3K27me3 compared to a random distribution of TSS (Fig. 3*A*). This is consistent with what is known about these histone markers and gene transcription (23, 24, 25, 26, 27, 28, 29, 30).

For both low-expressed (>1 TPM & < 10 TPM) and medium-high expressed genes (>10 TPM), the situation is reversed, with a greater overlap with H3K27ac compared to H3K27me3 (Fig. 3, *B* and *C*). Notably, the calculated Z-score is higher for the medium-high expressed genes, which is in line with expectations. The increased acetylation is required to maintain active transcription even in lowly expressed genes, and its magnitude is proportional to the level of gene expression (35, 36).

Next, we investigated the ability of ProOvErlap to reveal whether acetylated histone marks H3K27ac and H3K9ac are likely to colocalize with the histone modification reader BRD4 and the transcription factor SP1. BRD4 is known to bind acetylated lysines of histones (37, 35, 36), whereas SP1 normally binds DNA in the promoter regions of genes (38, 39). We started ProOvErlap with the following commands, using the merged genomic regions of BRD4 and SP1 as background:

- For BRD4:

“python3 prooverlap.py --input ENCFF130JVF_BRD4.bed --targets ENCFF864OSZ_H3K27ac.bed, ENCFF148UQI_ H3K9ac.bed --background BRD4_SP1_Background.bed --randomization 100 --mode intersect --genome genome.fa --orientation strandless --outfile BRD4_specs.txt”

“python3 prooverlap.py --input ENCSR583ACG _BRD4.bed --targets ENCSR000AKP_H3K27ac.bed, ENCSR000EVZ _H3K9ac.bed --background BRD4_SP1_Background.bed --randomization 100 --mode intersect –genome genome.fa --orientation strandless --outfile BRD4_specs_genomic_pos.txt --GenomicLocalization --gtf gencode.v45.annotation.gtf”

- For SP1:

“python3 prooverlap.py --input ENCFF553GPK_SP1.bed --targets ENCFF864OSZ_H3K27ac.bed, ENCFF148UQI_ H3K9ac.bed --background BRD4_SP1_Background.bed --randomization 100 --mode intersect --genome genome.fa --orientation strandless --outfile SP1_specs.txt”

“python3 prooverlap.py --input ENCSR991ELG_SP1.bed --targets ENCSR000AKP_H3K27ac.bed, ENCSR000EVZ_H3K9ac.bed --background BRD4_SP1_Background.bed --randomization 100 --mode intersect --genome genome.fa --orientation strandless --outfile SP1_specs_genomic_pos.txt --GenomicLocalization --gtf gencode.v45.annotation.gtf”

Using the merged peaks of BRD4 and SP1 as background, we can directly compare the two factors in terms of colocalization with the acetylated histone markers. Figure 3*D* shows that BRD4 is significantly more enriched than expected in regions overlapping with H3K27ac peaks, but not with H3K9ac. On the other hand, SP1 is preferentially colocalized with H3K9ac and not with H3K27ac (Fig. 3*E*). These observations suggest that BRD4 has a stronger affinity for the histone mark H3K27ac, which is involved in the maintenance of active enhancers. In contrast, H3K9ac is preferentially colocalized with SP1, a transcription factor known to bind to promoter regions.

The differential colocalization of BRD4 and SP1 with the two histone markers also appears to be associated with differential distribution in different genomic regions. Indeed, BRD4 is more likely to be found in intergenic and intronic regions, whereas SP1 is more likely to be found at promoter, TSS, 5′UTR, and exons (Fig. 3*F*).

### Evaluation of epigenetic differences between leiomyomas and normal myometrial tissue by analyzing H3K27ac-, MED12-, and CDK8-binding sites

Uterine leiomyomas or fibroids are the most common benign tumors of the female reproductive system. They are thought to affect more than 70% of adult women (40). Epigenetics and transcriptional changes play an important role in the development of leiomyomas, as shown by the frequent mutations in *MED12*, a subunit of the Mediator complex (41). Mediator complex is a 25 to 30 subunit transcriptional coactivator complex, and MED12 is required for the activation of Mediator kinase activity and RNApol II–dependent transcription (42, 43, 44). MED12 mutations trigger changes in enhancer architecture and transcriptional dysregulation typical of uterine leiomyomas (41). MED12 form a complex with cyclin C, CDK8, and MED13. Mutations in MED12 are responsible for a reduction of the cyclin C-CDK8 binding to chromatin at sites of active transcription (45, 46).

Therefore, we further investigated the efficacy of ProOvErlap in uncovering epigenetic and genomic associations using the publicly available ChIP-seq data from project PRJNA526864 (ENA accession number). Differences between uterine leiomyomas and normal myometrium were assessed using ProOvErlap (Fig. S3).

**Figure 4:**
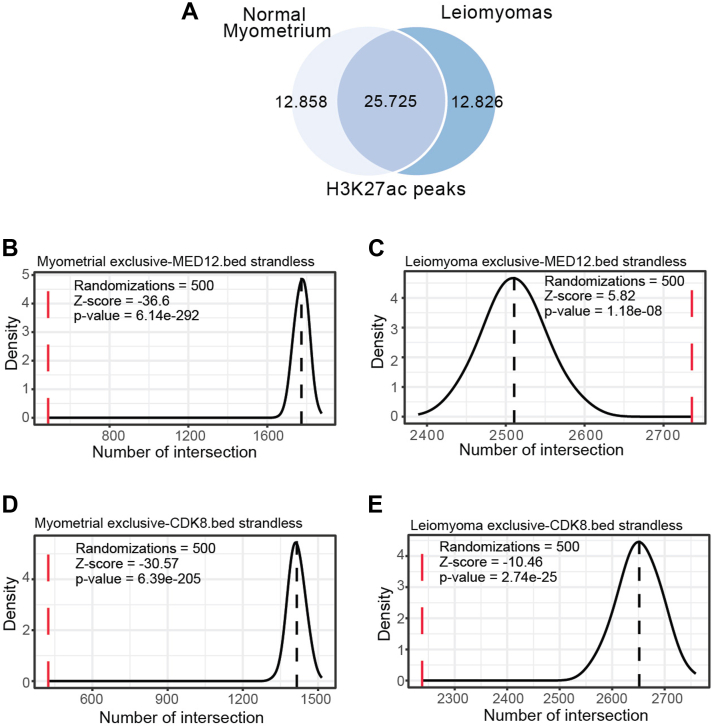
**ProOvEerlap and the epigenetic differences between leiomyomas and normal myometrial tissue**. *A*, Venn diagrams showing the overlaps and differences between H3K27ac ChIP-seq–enriched peaks obtained in myometrium and uterine leiomyoma. *B*, density plot showing the distribution of the mean distance from the random sampling compared to the real value. The *red dashed line* represents the actual number of overlaps between H3K27ac myometrium-exclusive peaks and targets: Myometrium-MED12-exclusive peaks. The *black dashed line* represents the mean value of overlaps of random H3K27ac peaks (leiomyoma and myometrium peaks combined) with the targets. Number of randomizations = 500. *C*, density plot as in (*B*). Here, leiomyoma exclusive peaks were analyzed. The *red dashed line* represents the real number of overlaps between H3K27ac leiomyoma exclusive peaks and the targets: leyomioma MED12 exclusive peaks. The *black dashed line* represents the mean of overlaps of random H3K27ac peaks (leiomyoma and myometrium peaks merged) with the targets. Number of randomizations = 500. *D*, density plot as in (*B*). Here myometrium-exclusive peaks were analyzed. The *red dashed line* represents the real number of overlaps between H3K27ac myometrial exclusive peaks and the targets: myometrium exclusive CDK8. The *black dashed line* represents the mean of overlaps of random H3K27ac peaks (leiomyoma and myometrium peaks merged) with the targets. Number of randomizations = 500. *E*, density plot as in (*B*). Here, leiomyoma exclusive peaks were analyzed. The *red dashed line* represents the real number of overlaps between H3K27ac leiomyoma exclusive peaks and the targets: leiomyoma exclusive CDK8 peaks. The *black* dashed line represents the mean of overlaps of random H3K27ac peaks (leiomyoma and myometrium peaks merged) with the targets. Number of randomizations = 500.

Since no differences were found between normal myometrium and leiomyoma with respect to the genomic distribution of MED12 and CDK8 in relation to H3K27 acetylation status (Fig. S3), we hypothesized that the specific local chromatin environment in myometrium and leiomyoma might contribute to the association of MED12 and CDK8 with chromatin. Therefore, we calculated the exclusive leiomyoma and normal myometrium peaks for H3K27ac, MED12, and CDK8 (Fig. 4*A*). As background, we merged the H3K27ac peaks of leiomyoma and normal tissues. Then we used ProOvErlap with the following command:

“python3 prooverlap.py –input leiomyoma _exclusive_H3K27Ac.bed –targets leiomyoma_exclusive_CDK8.bed, myoma_exclusive_CDK8.bed, leiomyoma_exclusive-MED12.bed, myoma_exclusive_MED12.bed –background H3K27ac_background_merged.bed --mode intersect --randomization 100 --genome genome.fa --orientation strandless --outfile leiomyoma_exclusive_H3K27ac.txt”

As shown in Figure 4*C*, specific MED12 peaks in leiomyoma samples are enriched in H3K27ac-marked leiomyoma-specific genomic regions and show less enrichment in normal myometrium (Fig. 4*B*). Both statistical tests yielded significant results, suggesting that MED12 enrichment in these genomic regions is not random and is higher than expected by chance in leiomyomas, whereas it is lower in normal myometrium. In contrast, in CDK8, the Z-score for leiomyoma-specific H3K27ac regions is higher than in normal tissue but remains below the randomization mean (Fig. 4, *D* and *E*). These results suggest that both the local epigenetic environment and MED12 mutations may differentially influence the interactions of MED12 and CDK8 with chromatin between leiomyoma and normal myometrium.

### Implementing ProOvErlap: Ranking analysis and leading regions identification

In genomic interval analysis, it is interesting to correlate certain genomic regions with a phenotype or a biological condition, for example, treated versus untreated samples or healthy versus diseased conditions. These analyses typically produce ranked lists of genomic regions ordered based on statistical metrics such as *p*-values, log2 fold changes, or other values. The ability to systematically analyze these ranked lists is critical to understanding whether genomic regions of high rank (*e.g.*, low *p*-values, high fold changes) consistently overlap or are in close proximity to target regions of interest. A major advantage of this approach is that it does not require an explicitly defined background set, as the background is already present in the BED input files. Furthermore, this method eliminates the need for arbitrary thresholds in the selection of relevant genomic regions, reducing potential biases that could influence the results. Inspired by the work of Subramanian et al. on gene set enrichment analysis, we have developed a similar approach for ProOvErlap (47). Our approach applies rank order analysis to overlap-based and distance-based evaluations.

For the overlap analysis, we evaluate the association between a ranked list of genomic regions and a predefined target set using an (optional weighted) ranking strategy. This approach quantifies the degree of enrichment of specific genomic features within a target set by calculating an enrichment score (ES). The analysis starts with a ranking of genomic regions based on an input score and then performs a random walk analysis, increasing the ES value if a region overlaps with at least one region in the target BED file and decreasing it otherwise. The maximum absolute ES value is used as the final enrichment value, indicating possible positive or negative enrichment. In addition, a leading analysis is performed to identify the regions that contribute most to enrichment and to provide a method for extracting the genomic regions associated with the biological context under study.

The input for the ranking analysis consists of a BED file, which must contain a score in the fifth column. The score field represents a quantitative measure of the association of each region with a phenotype, biological trait, or condition. This score can come from statistical analyses, such as significance values, differential expression studies, differential binding analyses, or other genome-based calculations that allow a score to be assigned to each region in the input file. Next, for each region in the BED input file, we calculate the overlap fraction, expressed as the number of nucleotides overlapping with at least one region in the target file divided by the length of the genomic region tested. To ensure comparability between input scores and overlap fractions, all scores are scaled between 0 and 1 using the min-max scaling technique. This scaling ensures that the input score has the same range (0–1) as the overlap fraction so that they can be consistently combined in weighted ranking analyses. After scaling, the genomic regions are ordered based on their input score. By default, the sorting is based on the input score only. Optionally, a weighted ranking method can be applied to prioritize regions with high proportions of overlap. In the weighted score method, the original score (here referred to as $S_{i}$) is adjusted using two factors:

(1) The overlap fraction $D_{i}$, which indicates the fractional overlap between an input region and target region.
(2) The scaled ranking $R_{i}$ (between 0 and 1), which indicates the relative rank of the region based on its original score.

To integrate these two factors, we first compute, for each score $S_{i}$, a weight $W_{i}$ as:

$$W_{i}=D_{i}\cdot \alpha +\left(1-R_{i}\right)\cdot \left(1-\alpha \right)$$

Where:

• $D_{i}$ is the overlap fractionᵢ and indicates the fraction of the region that overlaps with the target set
• $R_{i}$ is the relative rank of the region based on its original score.
• α is a tuning parameter that controls the relative influence of D and R. A higher α gives more importance to D, while a lower α gives more weight to R (α = 0.5 for equal importance).

Next, the original score $S_{i}$ is changed based on $W_{i}$, using a parameter w that determines how much the weighting affects the score:

If sorting is ascending:

$$S_{i}^{\prime }=S_{i}\cdot \left(\left(1-w\right)+w\cdot \left(1-W_{i}\right)\right)$$

If sorting is descending:

$$S_{i}^{\prime }=S_{i}\cdot \left(\left(1-w\right)+w\cdot W_{i}\right)$$

This aims to ensures that:

• In ascending sorting, if $D_{i}$ is high and $R_{i}$ is low (*i.e.*, a region with high overlap and a region with good ranking), the score $S_{i}$ decreases significantly, which further improves the ranking of the region in ascending sorting.
• In descending sorting, if $D_{i}$ is high and $R_{i}$ is low, the score $S_{i}$ increases, which further improves the ranking of the region in descending sorting.
• The parameter w controls how much $W_{i}$ influences the final score: if w is close to 1, the score is strongly adjusted; if w is 0, the score remains unchanged (the analysis relies on the original ranking based only on the input score).

By default, only input scoring is used, but users can enable weighting if they want to highlight regions that overlap heavily with the target set. Once the regions have been ranked (either by input score only or by weighted score), we perform a random walk enrichment analysis. This determines whether the ranked list has significant enrichment for regions that overlap with the target set at the start or end position of the ranked list. The following steps are used:

(1) The random walk starts with the first region in the ranking list.
(2) If a region overlaps with at least one region in the target file, the ES increases.
(3) If a region does not overlap with at least one region in the target file, the ES is reduced.
(4) The final ES value is the maximum absolute deviation from 0. We computed ES as follows:

$$p_{\text{hit}}=\sum_{i=1}^{N}\frac{1}{N_{h}}\left({\text{if}\;\text{region}}_{i}\text{is}\;\text{in}\;\text{the}\;\text{target}\;\text{else}\;0\right)$$

$$p_{\text{miss}}=\sum_{i=1}^{N}\frac{1}{N_{m}}\left({\text{if}\;\text{region}}_{i}\text{is}\;\text{not}\;\text{in}\;\text{the}\;\text{target}\;\text{else}\;0\right)$$

$$\text{ES}=p_{\text{hit}}-p_{\text{miss}}$$

finalES=max(∣ES∣)

where:

• N is the total number of regions.
• N_h_ is the number of regions that overlap at least one region in the target file.
• N_m_ is the number of nonoverlapping regions.
• The final ES value is the maximum absolute value of the ES score.

In addition to calculating ES, we perform a leading analysis to identify the regions that drive enrichment. The leading analysis is performed as follows:

(1) Determine the ES index of the peak corresponding to the highest absolute ES value.
(2) If the enrichment is positive, all regions before the peak are leading regions.
(3) In case of negative enrichment, all regions after the peak are leading regions.

These leading regions represent the major genomic intervals contributing to enrichment, making them key candidates for functional analysis. To determine whether the observed ES is statistically significant, we perform permutation tests. The input values ($S_{i}$) are randomly shuffled, and ES is recalculated multiple times (default: 100 permutations).

The null distribution of ES values is then used to calculate an empirical *p*-value as follows:

$$p_{\text{value}}=\frac{\sum \left(|\text{null}\_\text{ES}|\geq |\text{real}\_\text{ES}|\right)}{\text{num}\_\text{permutations}}$$

where:

• null_ES represents permuted ES values
• real_ES is the observed ES

A lower *p*-value indicates a stronger, nonrandom enrichment signal, while the sign of the ES value indicates a positive (overlapping regions are enriched at the beginning of the list) or negative (overlapping regions are enriched at the end of the list) enrichment. To increase efficiency, we parallelize the permutation tests using Python multiprocessing library. This allows multiple randomizations to be run simultaneously, which significantly speeds up computations when analyzing large genomic datasets or using a large number of permutations.

We also introduce a similar ranking analysis to test genomic distance. In the closest ranking test, we cannot simply derive an ES because distance is a continuous feature, while overlap can be categorized as a Boolean value. To account for this, we have slightly modified our approach. To assess whether the ranked genomic regions exhibit nonrandom proximity to the nearest target features (*i.e.*, genomic regions with higher scores are located near regions in the target file compared to regions with low scores or *vice versa*), we performed a statistical analysis based on the Kolmogorov–Smirnov test. This approach assesses whether the observed cumulative distribution of distances deviates significantly from a random expectation. For each genomic region in the input bed file, the closest region in the target bed file was determined. The absolute genomic distance $D_{i}$ between them was calculated, resulting in a list of distances:

$$D_{1},D_{2},\ldots ,D_{N}$$

where N is the total number of genomic regions in the input file. To assess how the distances are distributed in relation to their rank, we computed a normalized cumulative distribution ($S_{\text{real}}\left(i\right)$) of the real distances. Given a list of distances $D_{j}$, sorted by the rank of the associated genomic regions, the cumulative sum, up to position *i*, is defined as follows:

$$S_{\text{real}}\left(i\right)=\frac{\sum_{j=1}^{i}D_{j}}{\sum_{j=1}^{N}D_{j}}$$

where:

• $S_{\text{real}}\left(i\right)$ represents the cumulative sum of distances, up to region *i*, divided by the sum of all distances
• $D_{j}$ is the absolute distance of region *j* with respect to its closer feature in target file
• N is the total number of regions

To generate a null distribution, we randomly shuffled the distance values across the ranked regions so that the same distribution of distances was maintained but any potential relationship between ranking and proximity to target features was broken. For each shuffle, we calculated the corresponding cumulative distribution:

$$S_{\text{shuffle}}^{\left(k\right)}\left(i\right)=\frac{\sum_{j=1}^{i}D_{j}^{\left(k\right)}}{\sum_{j=1}^{N}D_{j}^{\left(k\right)}}$$

where:

• *k* represents an independent permutation

This procedure was repeated T times to generate an ensemble of random distributions. The mean normalized cumulative distribution was determined by averaging over all distributions as follows:

$$\left\langle S_{\text{shuffle}}\left(i\right)\right\rangle =\frac{1}{T}\sum_{k=1}^{T}S_{\text{shuffle}}^{\left(k\right)}\left(i\right)$$

In addition to ranking regions by their score, we introduced the possibility of using a weighted ranking to consider both the distance and the intrinsic score of the genomic region by using the same scaling approach as in the overlap analysis. First, we used the min-max scaling method to scale absolute distances between 0 and 1. Since we want to favor “small” distances in this case, we expressed the distance as follows:

$$1-D_{j}$$

We then applied the same normalization as for the Intersect test:

$$W_{i}=D_{j}\cdot \alpha +\left(1-R_{j}\right)\cdot \left(1-\alpha \right)$$

where:

• $D_{j}$ represents the scaled distance (in this case 1 − $D_{j}$) for feature *j* in the input file in relation to the nearest feature in the target file.
• $R_{j}$ is the scaled relative rank of the region based on its original score.
• α is a tuning parameter that controls the relative influence of D and R. A higher α gives more importance to D, while a lower α gives more weight to R (α = 0.5 for equal importance).

The original score $S_{i}$ is then changed based on $W_{i}$, using a parameter *w* that determines how much the weighting affects the score:

If sorting is ascending:

$$S_{i}^{\prime }=S_{i}\cdot \left(\left(1-w\right)+w\cdot \left(1-W_{i}\right)\right)$$

If sorting is descending:

$$S_{i}^{\prime }=S_{i}\cdot \left(\left(1-w\right)+w\cdot W_{i}\right)$$

This formulation ensures the following:

• In ascending sorting, if $D_{j}$ is high and $R_{j}$ is low (*i.e.*, a region that is very close and ranks well), the score $S_{i}$ decreases significantly, which improves the ranking of the region in ascending sorting
• In descending sorting, if $D_{j}$ is high and $R_{j}$ is low, the score $S_{i}$ increases, which further improves the ranking of the region in descending sorting.
• The parameter w controls how strong $W_{i}$ influences the final score: if w is close to 1, the score is strongly adjusted; if w is close to 0, the score remains unchanged.

To quantify whether the real distances differ significantly from expectations, we applied the Kolmogorov–Smirnov test between $S_{\text{real}}\left(i\right)$ and the mean of the shuffled distribution $\left\langle S_{\text{shuffle}}\left(i\right)\right\rangle$ using the “*ks_2samp*” function from the “scipy stats” Python library. The final ES was defined as the maximum absolute deviation between the real and the mean value of the expected distributions and was calculated as follows:

$$ES=\max|S_{\text{real}}\left(i\right)-\left\langle S_{\text{shuffle}}\left(i\right)\right\rangle |$$

Finally, the leading regions, that is, the regions that drive enrichment the most, were determined as follows.

(1) Determine the ES index of the peak corresponding to the position in the ranking associated with ES.
(2) If the enrichment is positive (*i.e.*, regions at the top of the list have higher distances with respect to target regions compared to shuffle distribution), all regions after the peak are leading regions.
(3) In the case of negative enrichment (*i.e.*, regions at the top of the list have lower distances with respect to target regions compared to shuffle distribution), all regions before the peak are leading regions.

**Figure 5:**
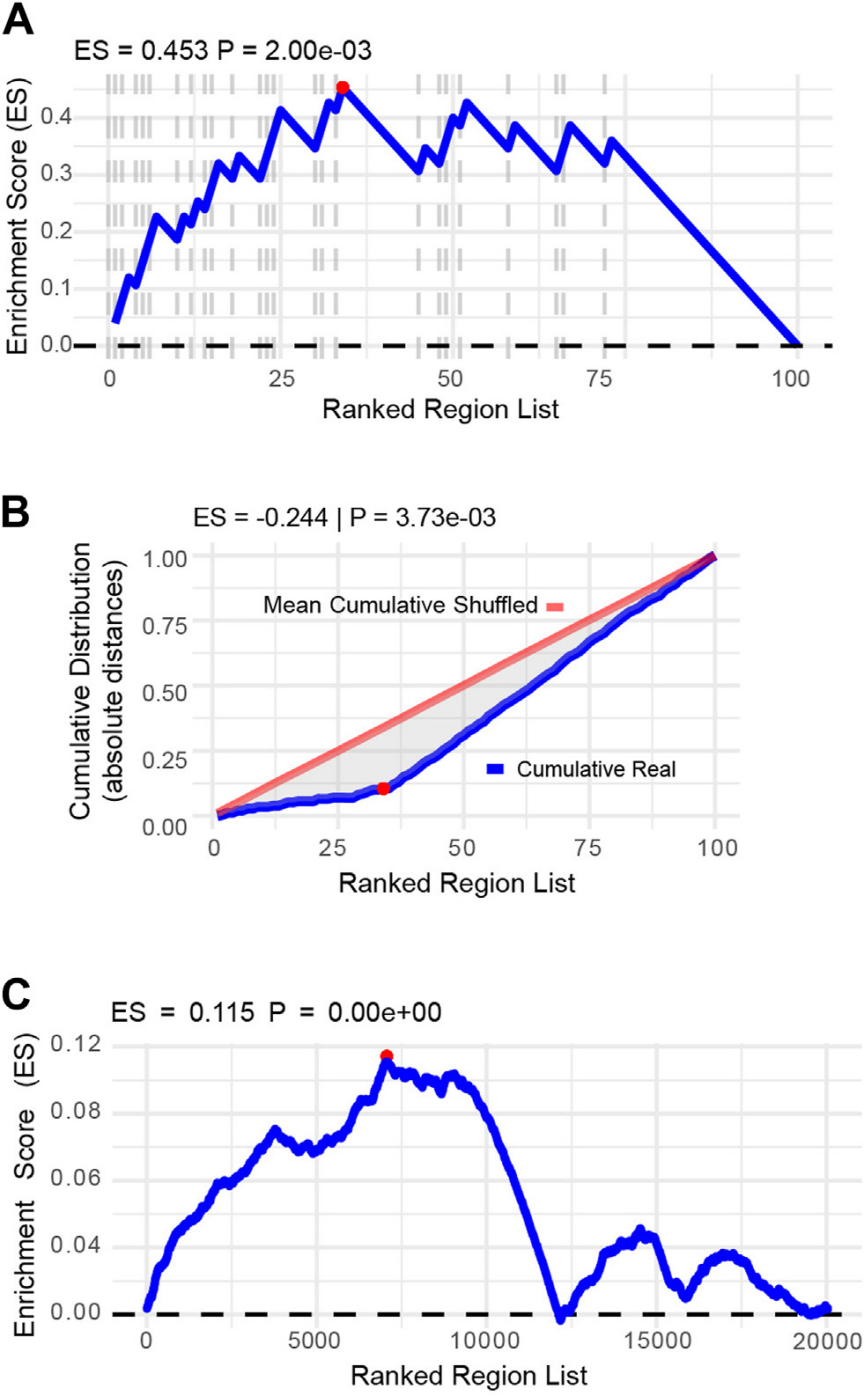
**Ranking analysis and leading regions identification**. *A*, enrichment plot for the ranked intersect test. The *blue* line represents the cumulative enrichment score (ES), while the *red* dot indicates the maximum ES. The *gray* dashed lines represent regions that overlap with at least one region in the target file and indicate their position in the ranking. The X-axis indicates the rank for each region in the input file, while the Y-axis indicates the ES. ES value and *p*-value are indicated in the title of the graph. All regions that lie before the point of maximum enrichment are labeled as “leading regions,” which represent the subgroup most responsible for the observed enrichment. *B*, enrichment plot for the ranked closest test. The *blue* line represents the cumulative real sum of the absolute distances for the ranked genomic regions in the input files with respect to the closest feature in the target file. The *red line* represents the mean cumulative sum of the absolute distances for the shuffled ranked genomic regions in the input files with respect to the closest feature in the target file. The X-axis indicates the rank for each region in the input file, while the Y-axis indicates the cumulative sum of absolute distances with respect to the closest region in the target file. We can observe a deviation of the real cumulative distribution from the mean cumulative distribution at the low ranks, indicating an enrichment of regions with lower distances at the top of the ranking. All regions that lie before the point of maximum absolute deviation are labeled as “leading regions,” which represent the subgroup most responsible for the observed enrichment. *C*, enrichment plot showing the cumulative enrichment score. Genes are sorted along the x-axis by decreasing log2 fold-change (day 6 *versus* day 0), while the cumulative enrichment score is shown on the y-axis. The *blue line* represents the running enrichment score for the ordered gene list, and the *red* dot highlights the point of maximum enrichment. All genes that lie before the point of maximum enrichment are labeled as “leading genes,” which represent the subgroup most responsible for the observed enrichment.

We evaluated the performance of the ranking analysis using synthetic datasets. For the overlap-based test, we created a ranked list of 100 genomic regions. The first 25 regions (*i.e.*, the regions at the top of the ranking list) were expected to overlap with at least one region in a synthetic target file and received a score from a normal distribution with a mean of 5 and an SD of ±3. The remaining 75 regions did not overlap with any of the regions in the target file and received higher scores on average (mean = 6, SD = ±3). In this way, we were able to artificially introduce enrichment for overlapping regions among the regions with lower scores. We then performed the ranking analysis by sorting the regions in ascending order of their scores so that the regions with lower scores appeared at the top of the list (Fig. 5*A*). As expected, we observed that regions with lower scores at the top of the ranking had a higher frequency of overlap with the target regions. In this case, the ES was 0.4 and the associated *p*-value was 0.002, indicating a statistically significant enrichment. These results confirm our expectation that regions with lower scores are more likely to overlap with the target dataset. We performed a similar analysis using a distance-based ranking test. Here, we created another set of 100 input regions. The first 35 regions were less distant from the closest feature in the target BED file, with a mean distance of 100 nucleotides (nt) and an SD of ±50 nt. These regions were also assigned lower scores (mean = 5, SD = ±3). The remaining 65 regions had a greater mean distance to the nearest target feature (mean = 150 ± 50 nt) and were assigned higher scores (mean = 6 ± 3). As before, we sorted the input regions in ascending order of their scores (Fig. 5*B*). We found that the cumulative distance of regions at the top of the ranking list increased more slowly than the mean permuted distribution of distances, suggesting that regions with higher rank (*i.e.*, lower score) are closer to the features in the target file. This suggests that our method can successfully identify feature enrichment based on proximity, highlighting the utility of score-based ranking for both overlap-based and distance-based enrichment analyses.

Finally, the ProOvErlap rank analysis was tested in a real biological context. Publicly available datasets containing both ATAC-seq and RNA-seq data obtained during *in vitro* differentiation of human B cells into plasma cells were analyzed. Data were obtained from three biological replicates using a well-established 13-days cytokine-driven differentiation protocol (48). In this experimental setting, we expect that upregulated genes should reside in open chromatin regions, as chromatin accessibility is strongly associated with the activation of gene expression (17). In order to test this hypothesis, we performed a differential gene expression analysis by comparing gene expression at day 6 *versus* day 0. Moreover, in order to test if genes characterized by higher fold changes are located in peaks unique to day 6, we identified the open chromatin regions private of day 6 compared to day 0. We then run ProOvErlap in descending rank test mode by sorting genes by decreasing log2(Fold Change) using the day 6 private ATAC peaks as target BED file. We observed significant positive enrichment, suggesting that genes with higher fold changes (*i.e.*, upregulated genes at day 6) tend to overlap more frequently with day 6–specific open chromatin regions (Fig. 5*C*). In particular, the highest ranked genes had the highest enrichment values, suggesting that a significant proportion of them are located in accessible chromatin. Conversely, after reaching the peak, the enrichment value gradually decreased across the entire ranking, suggesting that nonderegulated and downregulated genes generally have less overlap with open chromatin regions. These results support the hypothesis that chromatin accessibility plays an important role in controlling gene expression during B cell differentiation and activation (48).

## Discussion

In this manuscript, we have developed and described a new bioinformatics tool, called ProOvErlap, for analyzing BED files (obtained from NGS experiments) to reveal statistically significant associations between genomic regions. We tested and validated this proximity/overlap testing method on several publicly available datasets of NGS data, including RNA-seq, ATAC-seq, and ChIP-seq data.

ProOvErlap confirmed previous conclusions and provided new data and new hypotheses. This tool provides a comprehensive suite for performing enrichment analysis for genomic regions with flexible options for overlap, distance measurement, and content analysis. Its ability to handle different genomic contexts, along with randomization-based statistical tests, makes it a powerful resource for the analysis of genomic data represented in BED file format.

Existing methods for analyzing the overlap or proximity of genomic regions often rely on basic intersection-based approaches or predefined distance thresholds to measure the closeness between genomic intervals (49). Although these approaches are based on accurate measurements, they often lack statistical rigor and flexibility. ProOvErlap advances the field by integrating randomization-based statistical tests, enabling a more robust assessment of enrichment and proximity significance and allowing researchers to formulate hypotheses based on rigorous statistical significance thresholds. Furthermore, it ensures the use of a suitable background by considering all regions analyzed in an experiment. Additionally, when a consistent background is not available, ProOvErlap can generate an appropriate background by matching the chromosomal frequency and feature length of input regions, thereby providing a more accurate reference for statistical analysis.

While ProOvErlap offers powerful features for analyzing genomic regions in terms of enrichment and distance relationships as well as AT, GC content, and length, it is important to note some limitations. First, the tool is specifically designed to manage one-dimensional relationships between genomic intervals, such as the overlap and distance between regions on a linear genome. As such, it does not support the analysis of three-dimensional genomic relationships, including interactions such as chromatin loops, enhancer-promoter interactions, or the folding of the genome in three-dimensional space (24, 25, 50). Therefore, the tool cannot be used to analyze the spatial proximity of regions within the three-dimensional architecture of the nucleus nor can it capture interactions between distant genomic regions brought into proximity by chromatin folding or other structural mechanisms.

These limitations mean that the tool is best suited for studies involving standard one-dimensional genomic relationships, such as analyzing functional elements, gene regulation, or differential enrichment between conditions, but may not be suitable for studies that require the integration of three-dimensional genomic data or the study of chromatin interactions and folding.

With ProOvErlap, it is also possible to score genomic regions based on their overlap or proximity to a target set of regions. Regions can be ranked according to a score that reflects their association with a biological condition or phenotype. Enrichment is assessed by testing whether the highest or lowest ranked regions overlap with or are closer to the target regions than expected by chance. This application of ProOvErlap does not require a predefined background set or arbitrary thresholds. A flexible weighting system can adjust the rankings based on overlap percentage or distance. For overlap analysis, a random walk algorithm is used to detect enrichment patterns, while for distance analysis, the Kolmogorov–Smirnov test compares observed and randomized distance distributions. The regions responsible for enrichment are identified by a leading region analysis, which provides insight into the key regions contributing to the observed signal and form a candidate set for further biological validation.

In summary, feature overlap and proximity are fundamental concepts in bioinformatics that has wide applications in RNA-seq, ChIP-seq, and ATAC-seq experiments, among others, where understanding the one-dimensional spatial relationships between genomic features can reveal important regulatory mechanisms and disease associations. ProOvErlap provides a robust framework for assessing regions overlap and/or proximity and quantifying their significance using statistical methods such as randomization and permutations tests. By enabling researchers to systematically assess proximity and overlap in genomic datasets, this tool enhances our ability to identify biologically significant interactions and gain insights into gene regulation, protein–DNA interactions, and chromatin dynamics.

## Experimental procedures

### Datasets

The effectiveness of ProOvErlap in assessing feature proximity/overlap and testing statistical significance was evaluated using publicly available datasets from previous studies. The first dataset includes ATAC-seq and matched RNA-seq data from the K562 cell line, consisting of two biological replicates. The authors provide IDR-thresholded peaks in BED format for ATAC-seq, which annotate open chromatin regions, and RNA-seq data in a gene-to-FPKM matrix format to assess gene expression in both replicates. Open chromatin regions were extracted from IDR-thresholded BED files, while expressed genes were defined as those with expression greater than 1 FPKM in both replicates; all other genes were classified as nonexpressed. The background was defined as all genes measured in the experiment. These datasets are available on the ENCODE portal (ATAC-Seq data accession number: ENCSR483RKN and RNA-Seq data accession number: ENCFF556ISR). Moreover, we retrieved ChIP-seq data for three histone markers: H3K27ac (ENCODE accession number: ENCFF864OSZ), H3K27me3 (ENCODE accession number: ENCFF801AHF), and H3K9ac (ENCODE accession number: ENCFF148UQI). In addition, ChIP-seq data for the histone acetylation reader BRD4 (ENCODE accession number: ENCFF130JVF) and the transcription factor SP1 (ENCODE accession number: ENCFF553GPK) were obtained.

We also evaluate the tool on a publicly available dataset containing multi-omics data of RNA-seq, ChIP-seq, and HiC (GEO SuperSeries accession: GSE128242). We focused on the part of this study regarding the genome-wide H3K27Ac signal occupancy in fibroid tumors and matched normal myometrium tissue, as well as the genome-wide occupancy of CDK8 submodule factors CDK8 and MED12 (GEO SubSeries accession: GSE128230). To do this, we downloaded the following bed files available on GEO: GSM3667599_PT848-MYO-H3K27Ac.bed, GSM3667601_PT886-MYO-H3K27Ac.bed, GSM3667609_PT848-LEIO-H3K27Ac.bed, GSM3667611_PT886-LEIO-H3K27Ac.bed, GSM3667649_PT848—MYO-CDK8.bed, GSM3667650_PT886-MYO-CDK8.bed, GSM3667654_PT848--LEIO-CDK8.bed, GSM3667655_PT886-LEIO-CDK8.bed, GSM3667659_PT848—MYO-MED12.bed, GSM3667660_PT886-MYO-MED12.bed, GSM3667664_PT848—LEIO-MED12.bed, GSM3667665_PT886-LEIO-MED12.bed. With bedtools intersect, the corresponding ChIP-seq sample of the two patients (marked as PT848 and PT886) were intersected keeping only the consensus peaksets for the following test of this tool.

We also analyze publicly available datasets containing both ATAC-seq and RNA-seq data obtained during *in vitro* differentiation of human B cells into plasma cells (GEO SuperSeries accession: GSE219012). Data were obtained from three biological replicates using a well-established 13-days cytokine-driven differentiation protocol (48).

### Software implementation

The software is written in Python3 and takes advantage of the BEDTools software and the Python library pybedtools (15, 51). The software requires three BED files as input (only two if the “--generate_bg” flag is set or when ranking mode is used). BED files can be both six or more columns. The first BED file (referred to as the input file) defines the regions of interest for the enrichment analysis. The second BED file (called the target file) contains the genomic reference regions that are used to evaluate the enrichment of the input regions. The third BED file (the so-called background file) contains a set of genomic background regions from which the intervals in the input file are derived. For example, the input file should contain selected regions of interest, such as specific genes, regions differentially enriched between conditions, or other genomic features relevant to the study, while the background file may contain all genes within the given genomic context or a broader set of regions across the genome. Inclusion of a FASTA file is optional but recommended for sequence searching, content analysis, and gene structure assessment, as it provides the nucleotide sequences required to calculate base composition (*e.g.* AT and GC content) in the regions defined by the BED files and to generate the gene structure map.

The software can work in two modes: by analyzing overlap (--mode intersection) or by analyzing proximity (--mode closest). In “intersection” mode, the core of the analysis is to calculate the number of overlapping regions between the input and target regions. This is done using BedTools intersect, which identifies the genomic intervals in the input file that overlap with those in the target file (15, 51). The software allows customization of the overlap criteria using several parameters. The user can use the --exclude_intervals option to exclude certain genomic regions from consideration or the “--ov_fraction” option can be used to restrict the analysis to genomic regions with a defined minimum overlap fraction. The overlap can also be classified according to the strand direction. The analysis offers three modes: (i) “concordant” for features on the same strand, (ii) “discordant” for features on opposite strands, and (iii) “strandless,” where the strand direction is not considered. Importantly, a region from the input file that overlaps with multiple regions in the target file is counted only once to avoid overrepresentation and possible bias. After identifying overlapping regions, statistical enrichment is evaluated by comparing the observed overlap between the input and target files against a randomized background. The software performs 100 randomizations by default (the number can be adjusted), where genomic intervals are randomly selected from the background file and matched with the number of intervals in the input file. These randomizations generate a null distribution of overlaps that represents the random expected overlap. The observed number of overlaps is compared to this null distribution to assess the statistical significance of the observed enrichment. Significance is assessed using the Z-score, which is calculated as the difference between the observed number of overlaps and the mean number of overlaps from the randomized background divided by the SD of the random overlaps. The Z-score is then converted to a *p*-value based on a two-tailed Gaussian distribution. The Z-score can be interpreted as a measure of how far the observed overlap differs from the expected overlap under the null hypothesis of no enrichment (52).

Finally, the tool includes the --GenomeLocalization option, which allows additional analysis to determine whether the identified overlaps are in specific genomic regions, such as intergenic regions, promoters, TSSs, exons, introns, or UTRs. This option also requires the user to supply a gene annotation file in gtf format (--gtf option) used by the software to classify the different genomic regions. In addition, users can test for custom genomic localizations or other types of regions by creating a custom BED file and passing it to the script with the “-bed” option. In this case, the name column of the BED file is used to directly annotate the different regions included. This analysis is only available in Intersect mode.

In addition to spatial overlap, the tool performs randomization analyses of sequence content (AT and GC) and feature lengths (optional). These analyses follow the same randomization procedure as the overlap analysis but focus on comparing the observed AT/GC content or feature lengths with the randomization distributions derived from the background file.

In “closest” mode, the software performs a similar analysis, but instead of counting overlapping features, it measures the distance between features in the input file and their closest counterpart in the target file (15, 51). If a feature in the input file overlaps with a feature in the target file, the distance is counted as 0. If only the smallest distance is required and overlaps are irrelevant, the “--exclude_ov” flag can be used to remove all overlapping regions from the target file. Similar to the overlap-based analysis, the smallest distance can be evaluated under different strand conditions: (A) “concordant” for features on the same strand, (B) “discordant” for features on opposite strands, and (C) “strandless,” where the strand direction is not considered. In addition, the analysis can be restricted to upstream or downstream regions with respect to the features of interest using the flags “--exclude_upstream” or “--exclude_downstream.” The mean distance for the input features is calculated and compared to the mean distance from 100 randomizations (default, adjustable), and a Z-score and *p*-value were calculated to assess the significance of the observed distances compared to random background.

The software contains additional scripts for creating publication-ready visualizations that help to interpret the results. The “enrichment plot” allows the user to visualize the comparison between the observed data and the random distributions, while the heatmap provides a summary of all tested target files and/or genomic regions.

**Figure 6:**
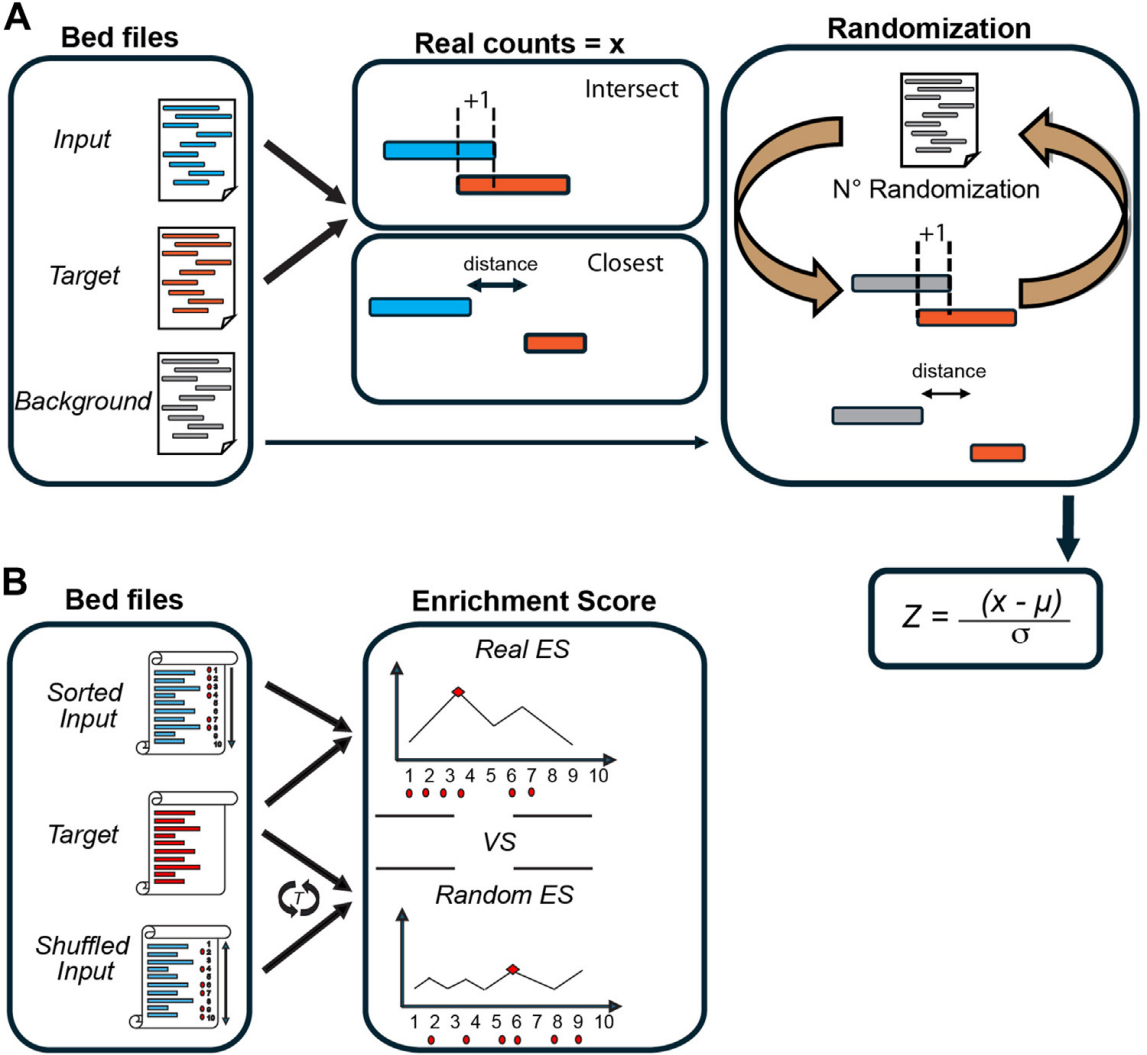
**The general workflow used in ProOvErlap.***A*, overview of ProOvErlap analysis workflow. The BED files Input and Target are required as well as an optional third file that represents the background (strongly recommended, alternatively a random background can be generated). The actual number of intersections or the mean distance between the genomic intervals in the input file in relation to the target file is evaluated. This value is then compared to a null distribution generated by randomly resampling the same number of genomic intervals from the BED background file and evaluating the number of overlaps or mean distances after N randomizations (100 by default). Finally, a Z-score is calculated to assess the significance level. *B*, overview of the ProOvErlap ranking analysis. Both input and target BED files were required. The input regions were ranked based on the “score” column in the input BED file. A random walk enrichment analysis was performed in which the enrichment score (ES) increases if the input region is overlapped with at least one region in the target file and decreases if no overlap was observed. The maximum absolute deviation from zero (highlighted as a *red dot*) was recorded as the final ES score. To assess statistical significance, the observed ES score was compared to a null distribution generated by permutation testing, in which the “score” values in the target file were randomly shuffled before calculating the random ES.

In cases where no suitable background file is available, the software can generate an artificial background by randomly selecting genomic regions. To do this, the chromosomal frequency distribution and length features of the input file are replicated to avoid positional bias. This process is controlled by the “--generate_bg” flag. In addition, the tool offers the possibility to exclude certain genomic regions from both the input file and the artificially generated background by using the option “--exclude_intervals.” A general overview of the process is shown in Figure 6*A* and *B*.

### ProOvErlap ranking analysis on real data

To test the analysis of enrichment by rank, publicly available dataset containing both ATAC-seq and RNA-seq data (GEO SuperSeries accession: GSE219012) were selected (48). Data were obtained from three biological replicates using a previously established 13-days cytokine-induced differentiation protocol (48). To test the rank enrichment analysis on real data, we focused on two differentiation time points: day 6 and day 0. Differential gene expression analysis was performed using the DESeq2 package on the publicly available gene count matrix, comparing gene expression between day 6 and day 0 (53). Gene coordinates (chromosome, start, and end) were retrieved using biomaRt, and a BED file was created in which the fifth column (score column) contains the log2 fold change (54). To define open chromatin regions specific to day 6, we identified ATAC-seq peaks that occur only under this condition using bedtools intersect (19). We then performed ProOvErlap rank enrichment analysis to determine whether genes that are upregulated at day 6 compared to day 0 (*i.e.*, those with a higher log2 fold change) are significantly enriched in the day 6–specific open chromatin regions. ProOvErlap was run with the default parameters, activating the rank order analysis mode (--RankTest) and using the descending sort order (default).

## Data availability

The tool is implemented in python3, distributed under the GNU General Public License (GPL) and available on Github at https://github.com/ngualand/ProOvErlap and PyPI at https://pypi.org/project/prooverlap/.

## Supporting information

This article contains supporting information.

## Conflict of interest

The authors declare that they have no conflicts of interest with the contents of this article.
