# Supplementary material for: ProOvErlap: Assessing feature proximity/overlap and testing statistical significance from genomic intervals
Source: J Biol Chem. 2025 May 8;301(6):110209. doi: 10.1016/j.jbc.2025.110209 (PMC12172997; doi:10.1016/j.jbc.2025.110209)
Supplement: Supplementary Material [file mmc1.docx]

**Supporting Information**

**ProOvErlap: Assessing feature proximity/overlap and testing statistical significance from genomic intervals**

Nicolò Gualandi, Alessio Bertozzo, Claudio Brancolini

### **
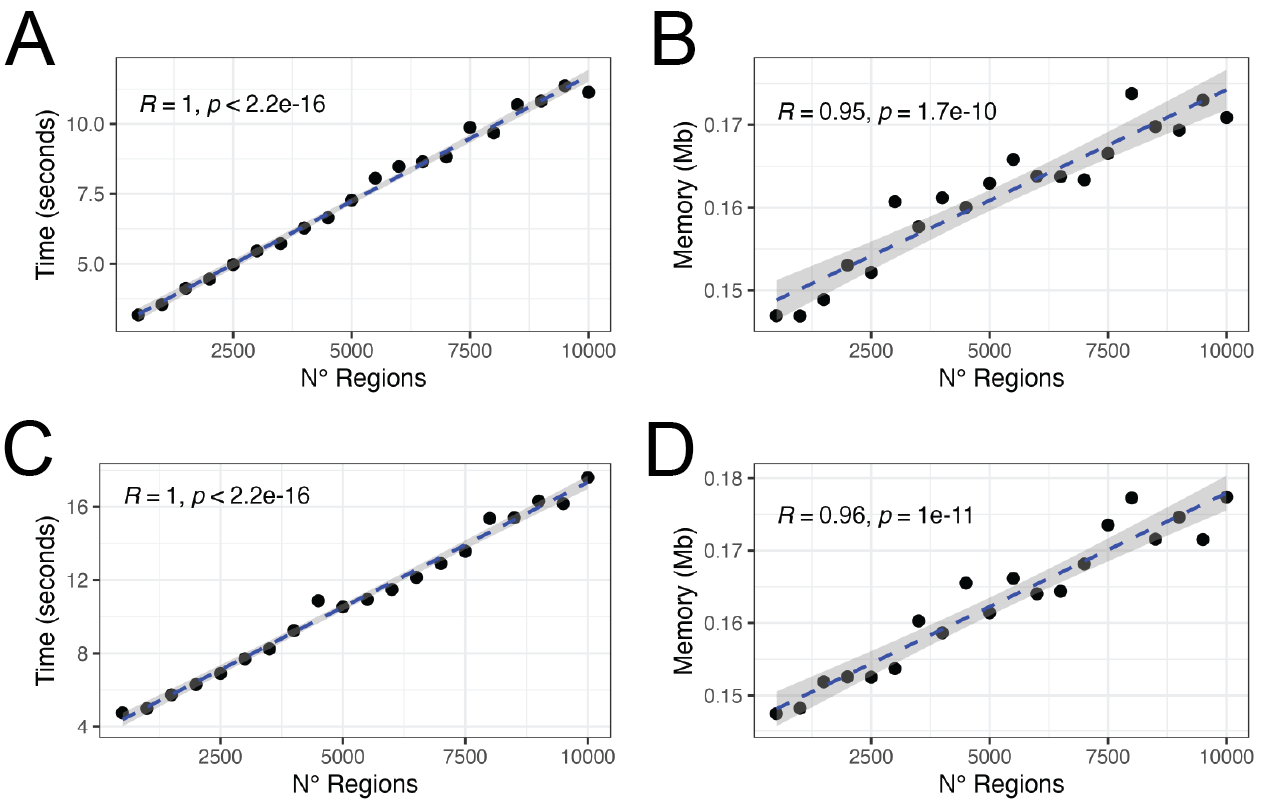
**

### **Figure S1. Running Time and Memory Usage Evaluation**

Execution time was recorded using the system's timing function (time), while memory usage was monitored using the “ps” process tracking command. Memory consumption was expressed as the maximum memory usage recorded during the execution of the software. All tests were performed on a system with a solid state drive (SSD) and a single processor.

A) Correlation between the number of regions (x-axis) in the BED input file and the runtime (y-axis) using the “intersect” test. The runtime is given in seconds. The Pearson correlation coefficient (R) and the p-value (p) are shown in the graph.

B) Correlation between the number of regions (x-axis) in the BED input file and the memory usage (y-axis) using the “intersect” test. Memory usage is given in megabytes (Mb). The Pearson correlation coefficient (R) and the p-value (p) are shown in the graph.

C) Correlation between the number of regions (x-axis) in the BED input file and the runtime (y-axis) using the “closest” test. The runtime is given in seconds. The Pearson correlation coefficient (R) and the p-value (p) are shown in the graph.

D) Correlation between the number of regions (x-axis) in the BED input file and the memory consumption (y-axis) using the “closest” test. The memory consumption is given in megabytes (Mb). The Pearson correlation coefficient (R) and the p-value (p) are shown in the graph.


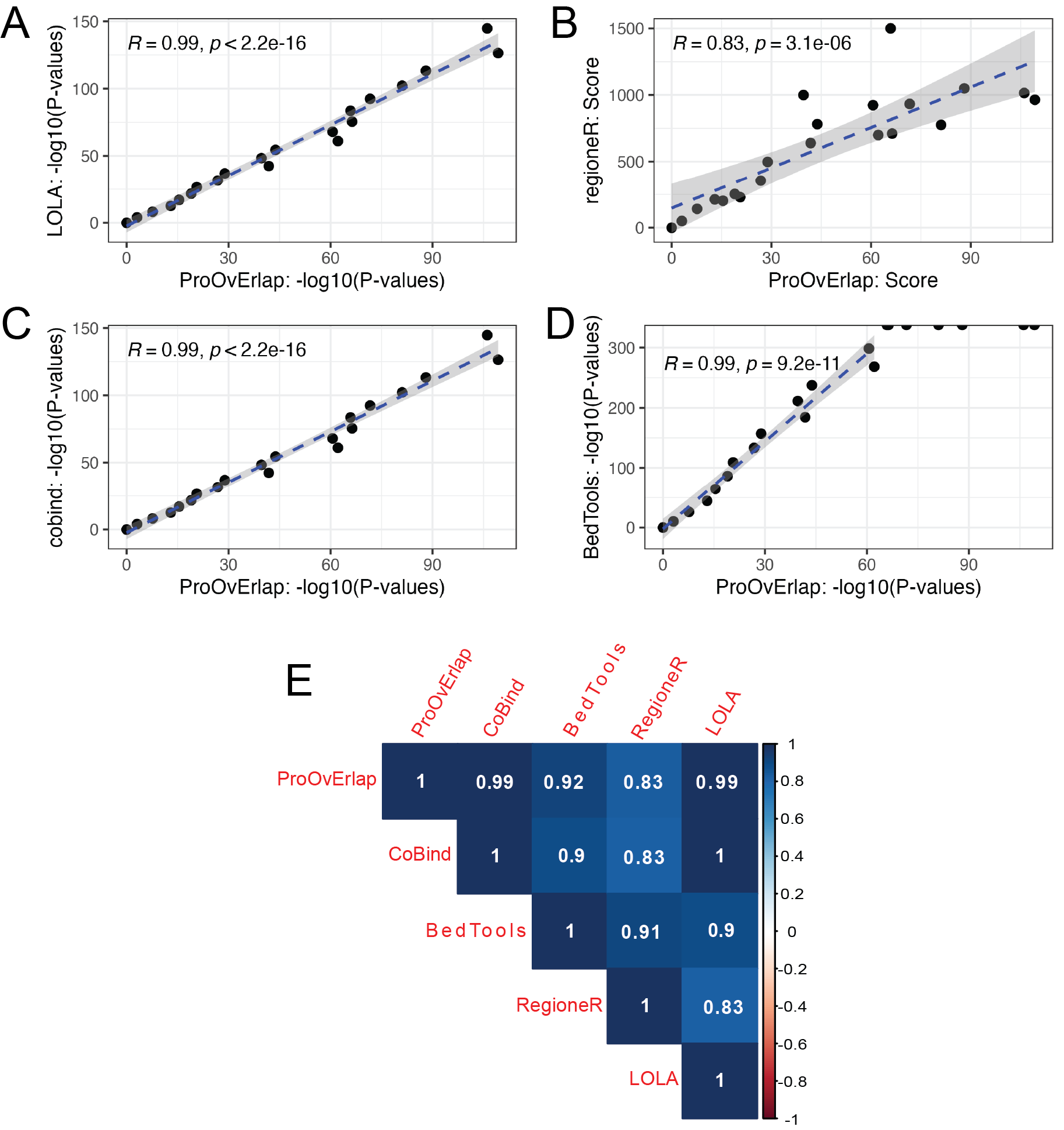


**Figure S2. Comparative analysis of ProOvErlap with LOLA, regioneR, CoBind, and Bedtools Fisher.**

The comparative analysis was assessed by the correlation of the -log10(p-value) for all tools except regioneR, which always yielded the same p-value for all tests. For regioneR, we used the reported score instead.

A) Correlation between the results (expressed as -log10(p-value)) for ProOvErlap (x-axis) and LOLA (y-axis) using a synthetic dataset. Both tools were run with the default parameters. The Pearson correlation coefficient (R) and the p-value (p) are shown in the graph.

B) Correlation between the results (expressed as -log10(p-value)) for ProOvErlap (x-axis) and RegioneR (y-axis) using a synthetic dataset. Both tools were run with the default parameters. The Pearson correlation coefficient (R) and the p-value (p) are shown in the graph.

C) Correlation between the results (expressed as -log10(p-value)) for ProOvErlap (x-axis) and CoBind (y-axis) using a synthetic dataset. Both tools were run with the default parameters. The Pearson correlation coefficient (R) and the p-value (p) are shown in the graph.

D) Correlation between the results (expressed as -log10(p-value)) for ProOvErlap (x-axis) and bedtools fisher (y-axis) using a synthetic dataset. Both tools were run with the default parameters. The Pearson correlation coefficient (R) and the p-value (p) are shown in the graph.

E) Heatmap of the correlation coefficients for all possible combinations. The color of the heatmap indicates the Pearson correlation coefficient. The exact Pearson correlation coefficients are given in each cell.

**
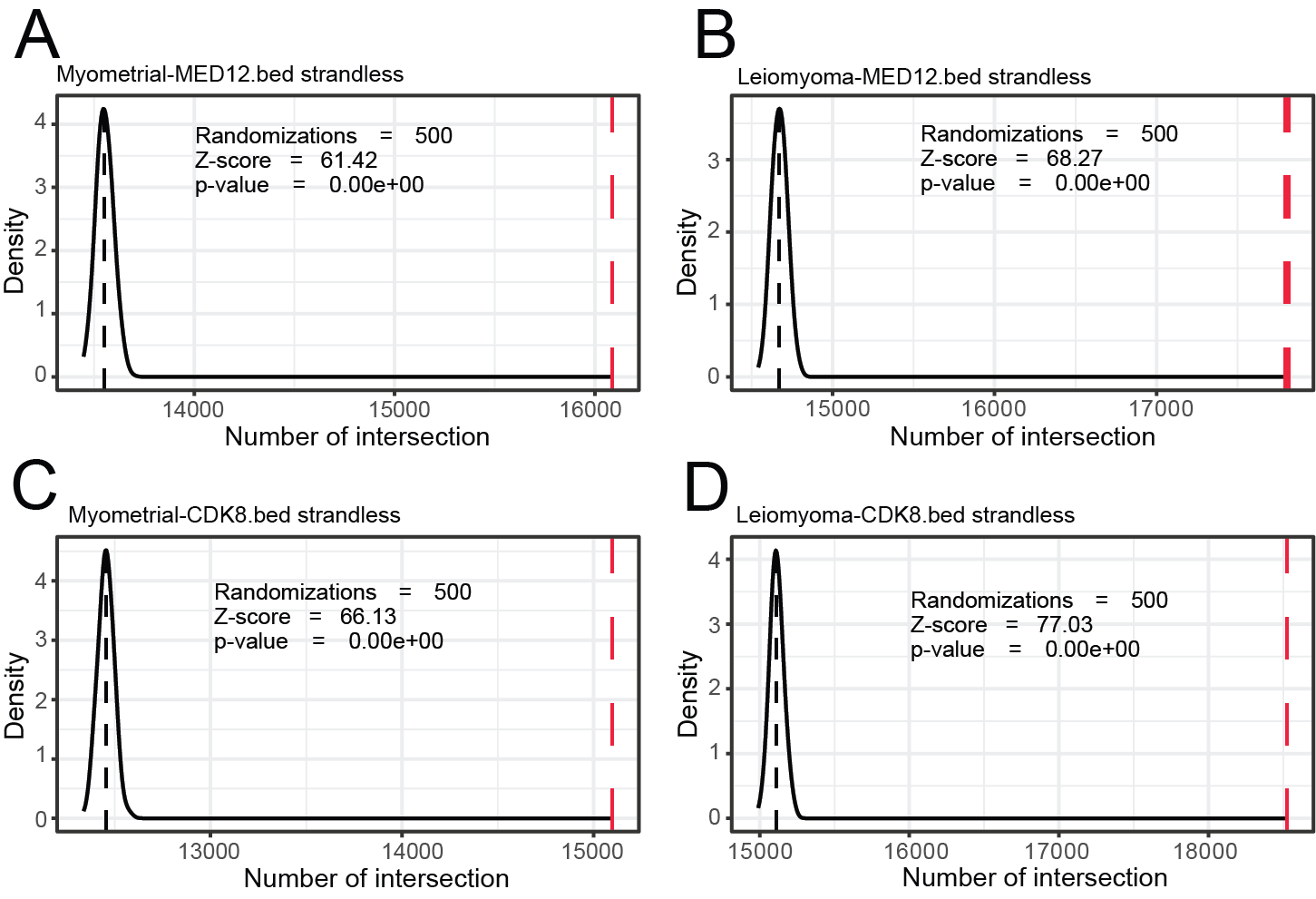
**

**Figure S3. ProOvErlap and the H3K27 acetylation in relation to MED12 and CDK8 distribution.**

Density plot showing the distribution of the mean overlap distance from the random sampling (black dashed line) compared to the real value (red dashed line). Number of randomizations = 500. Background: Leiomyoma and Myometrium H3K27ac peaks have been combined.

A) Overlaps of Leiomyoma H3K27ac peaks with Leiomyoma MED12 peaks.

B) Overlaps of Myometrium H3K27ac peaks with Myometrium MED12 peaks.

C) Overlaps of Leiomyoma H3K27ac peaks with Leiomyoma CDK8 peaks.

D) Overlaps of Myometrium H3K27ac peaks with Myometrium CDK8 peaks.

**Parameters and options recommendations**

**Input File (BED format) (--input):** The input must be a BED file with genomic regions, which can have 6 or more columns. For a file with more than 6 columns, only the first 6 columns are used for the analysis. If a ranking analysis is used, the 5th column of the BED file must contain the numerical scores for each genomic region. The BED file represents the regions of interest in a genomic context, e.g. peaks from a ChIP-seq experiment or other identified genomic features such as genes, promoters, enhancers or any kind of genomic interval that can be described by a chromosome, start and end position.

Target **File(s) (--target):** This parameter allows one or more BED target files to be specified in a comma-separated list. The target file represents the regions to be tested for overlap or proximity (i.e. closest distance). The analysis checks whether the regions from the input file overlap with at least one region in the target file, or calculates the distance to the closest feature in the target file. If several target files are specified, each target file is analysed independently.

**Background File (--background): The background file is a BED file containing genomic regions used for randomization. Each randomization selects a different subset of regions corresponding to the number of regions in the input BED file. This BED file is often a set of regions that represent the general genomic environment from which the regions in the input file originate. For example, if you are testing for significant binding in a ChIP-seq experiment, the background should represent all regions tested in the genome. The correct selection of this file ensures valid randomization and statistical comparisons. It should reflect the same distribution of genomic features as the input data.**

**Randomization (--randomization):** The number of randomization tests to be performed. Randomization is used to create a distribution of values from random data and allow comparison to assess whether the observed results are statistically significant. A higher number of randomizations (at least 100) results in more stable p-values. A lower number can reduce the computation time, but leads to less stable results. This process is important to determine whether the observed overlaps or distances differ significantly from what would be expected by chance. Recommended: > 100.

**Genome reference, FASTA format (--genome):** The genome reference file in FASTA format provides the sequence data required to analyze specific genomic features such as GC content, AT content and/or length. If the --test_AT_GC option is enabled, the script uses this file to extract the relevant content of the input regions. This file is important to understand how nucleotide composition and/or feature length can affect the analysis.

**Temporary directory (--tmp):** This is the directory where temporary files are stored during script execution. The directory is created automatically if it does not yet exist and is deleted again after successful execution. The temporary directory is only used to store intermediate files and is helpful when troubleshooting and correcting errors in the script.

**Output file (--outfile):** This parameter specifies the name of the output file in which the tabular results are to be saved. The results are saved in a tab-delimited format that can be easily opened and analyzed in various programs such as Excel, R or Python. The output file contains the analysis results for all input regions, including statistical evaluations such as p-values, enrichment values or other metrics.

**Output directory (--outdir):** This parameter specifies the name of the output directory in which additional tables are to be saved. This is useful for saving additional tables that are required for the following diagrams. It is recommended to use a new directory for each analysis.

**Orientation (--orientation):** This parameter determines how the strand direction is taken into account in the analysis. The options include:

- “strandless”: ignores strand direction and considers overlaps and closest features regardless of their strand.

- “concordant": Only features that are on the same strand as the target region are considered overlapping or closest.

- “discordant": Features located on opposite strands are considered overlapping or closest, while features located on the same strand are not considered.

**Overlap fraction (--ov_fraction):** This parameter specifies the fraction of overlap required between two genomic traits for them to be considered overlapping. The value is normally between 0 and 1, with 1 representing complete overlap. For example, a value of 0.5 means that a region is only considered overlapping if at least 50% of its length overlaps with the other region.

**Background generation (--generate_bg):** This option automatically generates a background file if no suitable file is available. It will attempt to create a background that reflects the same genomic distribution as the input data (e.g. matching chromosome frequencies and lengths). This is helpful if a specific background file is not readily available, but should be used with caution as it is generated based on the input data.

**Exclusion parameters:** These parameters allow certain genomic regions to be excluded from the analysis:

--exclude_intervals: a BED file containing regions to be excluded from both the overlap and closest feature analysis. Regions that overlap with this BED file are removed from the analysis.

--exclude_upstream: Excludes upstream regions from the closest feature analysis so that only downstream regions are considered as closest features.

--exclude_downstream: Excludes downstream regions when analysing the closest feature, i.e. only upstream regions are considered.

The excluding parameters follow the rule specified by the “Orientation” parameters.

**Additional feature tests:** These options enable additional tests for the input regions:

--test_AT_GC: Calculates the AT and GC content of each region in the input file. This helps to understand the nucleotide composition of the analysed regions.

--test_lengths: Calculates the length distribution of the regions in the input file, which can be useful for comparing shorter and longer regions.

**Genomic localization analysis (--GenomicLocalization):** This test evaluates the enrichment of overlapping regions relative to known genomic structures (such as introns, exons, UTRs). It requires a GTF or custom BED file with gene annotations and provides information on whether certain input regions that overlap with target regions are enriched in certain genomic contexts. The annotation file must be specified with either the –gtf or –bed option. If the –bed option is used, the fourth column in the BED file is used to group regions of the same group (i.e. exons, introns or even custom names). If the –gtf option is specified, the script automatically extracts promoters, UTRs, exons and introns from the GFT file.

**Ranked mode (--RankTest):** This option enables rank-based analysis, where regions are ranked based on their scores. This method can be useful to prioritize certain regions over others based on predefined metrics. When Ranked mode is enabled, the background file is not required.

**Sorting order (--Ascending_RankOrder):** This parameter determines the order in which the regions are ranked and sorted. If it is activated, ascending is set to True so that the regions are sorted in ascending order of their scores. If it is not activated, the regions are sorted in descending order by default.

**Weighted ranking (--WeightRanking):** When this option is enabled, the ranking of regions is adjusted based on a weighted score. The weighting can be calculated based on factors such as distance to the target region or overlap percentage.

**Weighting parameters:** These parameters are used to control how much the different factors affect the final score:

--alpha: setting parameter that controls the relative influence of overlap proportion/distance and relative rank. A higher α gives more importance to overlap proportion/distance, while a lower α gives more weight to relative rank (α = 0.5 for equal importance). Recommended: 0.5 (equal weighting of distance/overlap and relative rank).

--w: A weighting value between 0 and 1 that determines how much the calculated weighting (W) influences the final score. A value closer to 1 gives more weight to the adjustment, while a value closer to 0 leaves the score unchanged. Recommended: < 0.25.

Weighted ranking and weighting parameters should be used with caution as they can significantly influence the final ranking of the genomic regions. Choosing a weighting factor (w) that is too high can drastically alter the initial ranking by disproportionately favoring regions with low distance or high overlap. To maintain biological relevance and interpretability, it is critical to choose a weighting parameter that balances the contribution of distance and overlap without overly amplifying their effects. Overweighting these factors can lead to a biased ranking that does not accurately reflect the underlying biological signal, but instead prioritizes regions based solely on their spatial proximity or degree of overlap. Therefore, careful parameter selection and sensitivity analyzes are recommended to ensure that the weighted ranking improves, rather than biases, the identification of biologically significant genomic regions.

**Parallelization (--**thread**):** This parameter defines the number of threads to be used for parallel processing. The use of several threads can speed up the analysis, especially with large data sets or numerous randomizations. Recommended: all available threads.
